# Statistical Test of Expression Pattern (STEPath): a new strategy to integrate gene expression data with genomic information in individual and meta-analysis studies

**DOI:** 10.1186/1471-2105-12-92

**Published:** 2011-04-11

**Authors:** Paolo Martini, Davide Risso, Gabriele Sales, Chiara Romualdi, Gerolamo Lanfranchi, Stefano Cagnin

**Affiliations:** 1 CRIBI Biotechnology Centre, University of Padova, via U. Bassi 58/B, 35121 Padova, Italy; 2 Department of Biology, University of Padova, via U. Bassi 58/B, 35121 Padova, Italy; 3 Department of Statistical Science, University of Padova, via C. Battisti 241, 35121 Padova, Italy

## Abstract

**Background:**

In the last decades, microarray technology has spread, leading to a dramatic increase of publicly available datasets. The first statistical tools developed were focused on the identification of significant differentially expressed genes. Later, researchers moved toward the systematic integration of gene expression profiles with additional biological information, such as chromosomal location, ontological annotations or sequence features. The analysis of gene expression linked to physical location of genes on chromosomes allows the identification of transcriptionally imbalanced regions, while, Gene Set Analysis focuses on the detection of coordinated changes in transcriptional levels among sets of biologically related genes.

In this field, meta-analysis offers the possibility to compare different studies, addressing the same biological question to fully exploit public gene expression datasets.

**Results:**

We describe STEPath, a method that starts from gene expression profiles and integrates the analysis of imbalanced region as an *a priori *step before performing gene set analysis. The application of STEPath in individual studies produced gene set scores weighted by chromosomal activation. As a final step, we propose a way to compare these scores across different studies (meta-analysis) on related biological issues. One complication with meta-analysis is batch effects, which occur because molecular measurements are affected by laboratory conditions, reagent lots and personnel differences. Major problems occur when batch effects are correlated with an outcome of interest and lead to incorrect conclusions. We evaluated the power of combining chromosome mapping and gene set enrichment analysis, performing the analysis on a dataset of leukaemia (example of individual study) and on a dataset of skeletal muscle diseases (meta-analysis approach).

In leukaemia, we identified the Hox gene set, a gene set closely related to the pathology that other algorithms of gene set analysis do not identify, while the meta-analysis approach on muscular disease discriminates between related pathologies and correlates similar ones from different studies.

**Conclusions:**

STEPath is a new method that integrates gene expression profiles, genomic co-expressed regions and the information about the biological function of genes. The usage of the STEPath-computed gene set scores overcomes batch effects in the meta-analysis approaches allowing the direct comparison of different pathologies and different studies on a gene set activation level.

## Background

In the last decades, microarray technology has seen such an explosion of applications as to become a standard tool in biomedical research. It has allowed the discovery of many prognostic genome markers related to the development of pathologies [1-6]. The spreading process has brought a dramatic increase in the number of publicly available datasets [7-9].

Given the high-throughput nature of microarrays, statistical and bioinformatic methods were required to analyse such large amounts of data. Initial studies were focused on the identification of differentially expressed genes and their significance in many experimental designs (gene by gene approach). This analysis is time-consuming and sometimes ineffective because derived gene lists have to be interpreted, searching for patterns of genes that have similar function or are involved in particular processes [10]. This approach revealed that genes that are identified as differentially expressed often do not correlate with the phenotype under investigation. Furthermore, their consistency often decreases when different studies on the same biological issue are compared (meta-analysis approach) [11].

Meta-analysis may be broadly defined as the quantitative review and synthesis of the results of related but independent studies [12]. Different groups demonstrated its applicability to microarray data. Rhodes [13] applied meta-analysis to combine four datasets on prostate cancer to determine genes that are differentially expressed between clinically localized prostate and benign tissue. Parmigiani [14] performed a cross-study comparison of gene expression for the molecular classification of lung cancer. Park and Stegall [15] combined publicly available datasets and their own microarray datasets to investigate the detection of cytokine gene expression in human kidney. Meta-analysis studies clearly showed that the different lists of differentially expressed genes from different studies overlap poorly due to the complicated experimental variables embedded in array experiments. This suggests that a pathway/gene set-based approach could improve the performance of this type of comparison [16].

To improve microarray data analysis, the first tools developed were based on the integration of external genomic information such as gene location [17-19], ontological annotations [20-23] or sequence features [24].

Several methods were devised to analyse gene expression as a function of physical location of genes on chromosomes. These approaches, collectively referred to as "chromosome mapping", were applied to microarray data of cancer studies. The studies identified regions with transcriptional imbalances that reflected large chromosomal aberrations typical of such pathologies. Examples of these applications are the Locally Adaptive statistical Procedure (LAP) [17] and the MicroArray Chromosome Analysis Tool (MACAT) [18]. LAP was applied to compare gene expression data of acute myeloid leukaemia (AML) with and without trisomy on chromosome 8. LAP correctly identified the over-expressed region on chromosome 8 of patients where DNA amplification was present. MACAT was applied to compare T and B lymphocytes from patients with acute lymphoblastic leukaemia (ALL), identifying a marked over-expression of the region that contains genes of class II major histocompatibility complex (MHCII, chr:6p21.33-6p21.2) in the B lineage. Recently, a chromosome mapping approach based on the Haar Wavelet transformation (Chromowave) [19] was applied to a dataset of Huntington's disease. The study demonstrated that the aberrant interaction between the mutant huntingtin protein and its transcriptional co-activators, such as histone acetyltransferase, leads to large areas of transcriptional imbalances [25].

A more popular method for the integration of gene expression profiles with additional information is based on ontological and pathway annotations and is called Gene Set Analysis (GSA). This approach evaluates gene expression profiles among groups of related genes (gene sets), seeking coordinated changes in the expression levels of subsets of gene members. Usually, GSA has three main steps: a) computing associations of each expression pattern with a phenotype; b) computing enrichment scores for analysed gene sets; c) computing the global p-value and q-value for every tested gene set based on the appropriate permutation test. Several implementations of the GSA approach are now available, such as the algorithms developed by Subramanian (Gene Set Enrichment Analysis; GSEA) [22], Tian (sigPathway) [23], Efron (with the improvement based on the use of the maxmean statistic for summarizing gene sets) [26] and Goeman with Global Test [21].

Recently, Szabó [27] combined mRNA and comparative genome hybridization results, revealing that the major pathogenetic pathways involved in adrenocortical tumours are related to regions with aberrant gene expression. This work is an example of how the integration of different genomic information is useful to gain new insight into a pathology by exploiting available datasets. We believe that an important shortcoming of Szabó's described method is that it is based only on differentially expressed genes thus defining a strict cut-off without considering the actual level of expression.

Here, we propose a new procedure, STEPath (Statistical Test of Expression Pattern), that scores and integrates chromosomal region activation as an *a priori *step before performing GSA. The result of this analysis is a global expression value of gene sets weighted by chromosomal region activation. The plasticity of the chromosome architecture was recently debated due to the identification of transcription factories [28,29], but there are no bioinformatic algorithms that consider this aspect in the gene set analysis. Three public datasets were tested. We demonstrated that the combination of gene expression profiles, chromosome mapping and gene set analysis produced gene set scores suitable to compare different studies in a meta-analysis approach.

## Results and Discussion

### STEPath Algorithm

We implemented a new gene expression analysis method that takes into account i) the activation or repression of genes in chromosome regions [30] and ii) the evidence that intensive transcription at one locus frequently spills over in physically adjacent loci [31]. The STEPath algorithm allows scoring and integrating these aspects of gene regulation (i and ii; Step 1) before performing gene set analysis (Step 2). Gene set scores from step 2 can be used for meta-analysis studies (Step 3).

#### Step 1

To integrate physical locations of genes, STEPath measures the association of a gene expression profile with a phenotype (e.g., Significance Analysis of Microarrays (SAM) statistics [32]), rescaling it on the expression levels of the neighbour genes. We analysed each gene in relation to the y closest up- and down-regulated genes. Using a permutational approach, we tested the following hypothesis: *H*^*0*^, the region did not show differential expression; *H*^*1*^, the region is differentially expressed.

#### Step 2

After the computation of chromosome profiles using significant regions, STEPath performs a gene set analysis using SAM statistics [32] smoothed according to the chromosome profiles. We defined this step as a gentle integration of the chromosome profile because the smoothing process does not penalize gene scores; instead, it attributes to gene members of differentially expressed regions an additional score proportional to their own SAM score and to the local profile. This method enhances particular signals along chromosomes that are buried in the background due either to sample or technical heterogeneity that could profoundly affect microarray reproducibility.

Using the smoothed statistic, an up- and down-regulation value for every gene set was calculated. As GSA relies on the quality of annotation and dimension of gene sets, it is possible that the signal of a small group of coordinated genes becomes lost in non-specific signals. If this could be an analysis limitation, we increased GSA power by adopting two approaches: a) we analysed the most-used database repositories for gene sets, and b) we extracted portions of pathways showing coordinated expression.

a) We have compiled gene sets from Gene Ontology (GO) [33], Kyoto Encyclopedia of Genes and Genomes (KEGG) [33-36], BioCyc [37], BioCarta [38], SuperArray [39] and WikiPathways [40]. The resulting database includes Cellular Component, Molecular Function and Biological Process (GO derived) sets, manually curated functional pathways from BioCarta, metabolic pathways from KEGG and specific pathways for quantitative Real Time PCR (qRT-PCR) and microarray experiments from SuperArray. If different annotations of the same biological aspect may produce redundancies, they also retain specific differences and provide both robustness and specificity when correlated simultaneously to a phenotype (see Additional file 1; Figure S1).

b) According to Efron [26] and later confirmed by Song [41], splitting up and down portions of gene sets improves the statistical power of approaches where the mean of a statistic is used to score gene sets.

Up- and down-regulation scores were independently tested for significance using a gene-based permutation approach. We tested the null hypothesis, *H*^*0*^, that the gene set shows the same pattern of association with the phenotype compared to the rest of the genes. The q-values were computed using the Benjamini Hochberg algorithm [42].

#### Step 3

In meta-analysis studies, step 3 primarily aims to determine if the results from one study are confirmed in other independent studies.

For an individual study, the STEPath procedure (Step 1 plus Step 2) produces a list of gene sets with summarization values (*Gup *and *Gdown*, see Methods) and an associated q-value. Different pathologies can then be directly compared using the gene set summarization values produced for individual studies.

### Individual analysis of Leukaemia Dataset

We tested STEPath on an expression profile dataset of patients affected by Acute Lymphoblastic Leukaemia (ALL; 16 with and 90 without translocation of the Mixed-lineage leukaemia (MLL) gene).

Raw expression data (CEL files) were downloaded from the GEO database (GEO series ID: GSE14062), processed using a gene-based custom Chip Definition File (CDF) [43] to better define the chip [44], and normalized using the Robust Multichip Average (RMA) expression summary [45]. We recovered expression values for 15,953 genes. Using STEPath, we directly compared ALL with (ALL/MLL+) and without MLL translocation (ALL/MLL-), seeking evidence specific for MLL translocation. Genes without chromosomal location information and genes on chromosome Y were filtered out because paucity of gene expression data precludes the application of chromosome mapping.

This dataset was used to analyse the performance of the main modules implemented in STEPath: chromosome mapping (Step 1) and gene set analysis (Step 2).

#### Step 1

**Figure 1:**
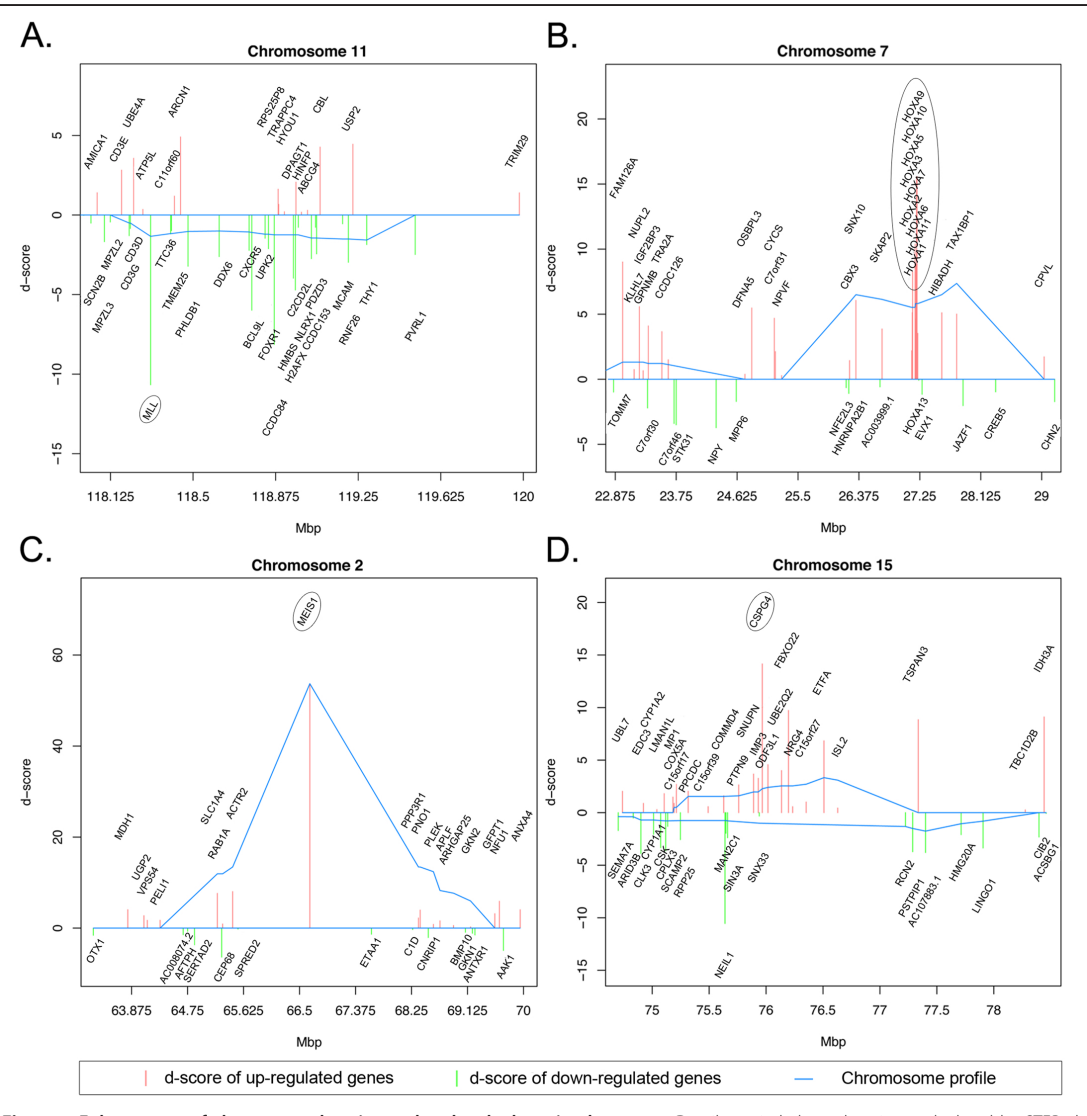
**Enlargement of chromosomal regions related to leukaemia phenotype**. Details on imbalanced regions calculated by STEPath chromosome mapping. Blue line represents chromosome profile; red and light green bars represent gene statistic values (d-score). A. Enlargement of the region of chromosome 11 containing the MLL gene (gene highlighted by the circle). B. Enlargement of the region between 20 and 32 Mbp of chromosome 7. This region corresponds to the localization of the HOX gene cluster (cluster highlighted by the circle). C. Enlargement of the region between 51 and 75 Mbp of chromosome 2 corresponding to the MEIS1 windows (gene highlighted by the circle). D. Enlargement of the region of chromosome 15 containing the NG2 gene (gene highlighted by the circle).

Using our implementation, we were able to identify a spectrum of possible imbalanced regions across all chromosomes (see Additional file 1; Figure S2). We identified the down-regulation of the region that contains the MLL gene (Figure 1A; Additional file 2; Table S1). MLL is characterized by a chromosome rearrangement, disrupting its correct localization and transcriptional regulation [46].

Our method highlights interesting imbalanced regions that contain genes involved in ALL pathology: 1) the region containing the Homeobox genes (HOX cluster) on chromosome 7 (Figure 1B; Additional file 2; Table S2), and 2) the region containing Meis homeobox 1 (MEIS1) on chromosome 2 (Figure 1C; Additional file 2; Table S3).

As discussed by Zangrando [47], HOX genes and MEIS1 are up-regulated in ALL, representing a discriminant signature that separates ALL/MLL+ from ALL/MLL-. Another gene involved in the discrimination between ALL/MLL+ and ALL/MLL- patients is the chondroitin sulfate proteoglycan 4 (CSPG4/NG2). NG2 encodes for a transmembrane protein located in the leukaemic cell membrane and proposed as a marker for rapid classification of ALL with MLL translocation [48]. Zangrando [47] used SAM and Predicted Analysis of Microarray (PAM) [49] to identify this discriminant gene. Our method pointed out this gene as well, evidencing the goodness of our algorithm (Figure 1D; Additional file 2; Table S4).

We compared our results with the most used approaches to detect imbalanced regions, namely LAP [17] and MACAT [18]. We ran these algorithms using the suggested number of permutations (10,000 for LAP and 1,000 for MACAT, see Additional file 1; Figure S3 and S4) and also with our settings (100 permutations, see Additional file 1; Figure S5 and S6). Different numbers of permutations did not result in relevant differences in the detected regions.

The comparison between LAP results (see Additional file 1; Figure S3) and STEPath chromosome mapping (see Additional file 1; Figure S2) shows that our approach identifies more regions than LAP. We decided to preserve information since this is not an independent procedure, but it is later piped into a gene set analysis. Even if LAP identifies a smaller number of imbalanced regions, these span larger chromosome portions, such as those on chromosome 3 (see Additional file 1; Figure S3), causing difficult interpretation of the results.

**Figure 2:**
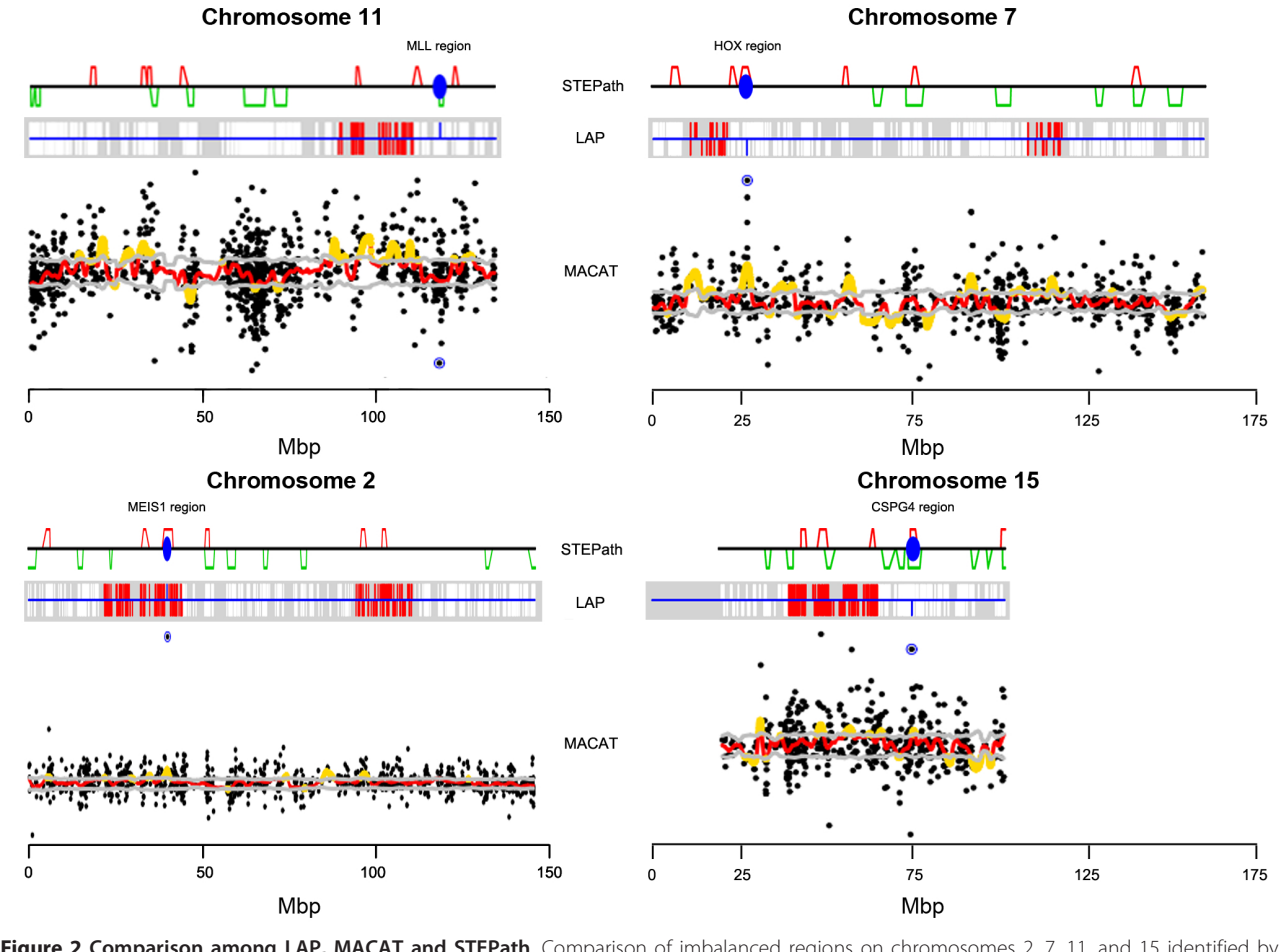
**Comparison among LAP, MACAT and STEPath**. Comparison of imbalanced regions on chromosomes 2, 7, 11, and 15 identified by LAP, MACAT and STEPath. LAP procedure fails to identify as imbalanced the MLL region on chromosome 11, the HOX genes cluster on chromosome 7, and the CSPG4 containing region on chromosome 15, while identifies MEIS1 region on chromosome 2. MACAT also fails to evidence the MLL region on chromosome 11.

In contrast with LAP, our procedure limits imbalanced regions to small portions of interest that are easier to visualize and relate to the studied phenotype. Indeed, the LAP procedure fails to identify as imbalanced the MLL region on chromosome 11 and the HOX genes cluster on chromosome 7 (Figure 2 and Additional file 1; Figure S3). LAP identified MEIS1 region on chromosome 2, but this region spans about 45 Mbp (from ~30 Mbp to ~75 Mbp, Figure 2 and Additional file 1; Figure S3). Our algorithm reduces the region to ~11 Mbp (from 62 Mbp to 73 Mbp), focusing on truly disease-related genes. These results demonstrate that our algorithm seems to be more sensitive than LAP in the identification of important imbalanced regions involved in ALL.

Furthermore, our procedure reaches a greater sensitivity than MACAT in the detection of regions on chromosome 2, 7, 11 and 15 (Figure 2). The profile of chromosome 11 produced with MACAT (see Additional file 1, Figure S4) is greatly perturbed, and it is difficult to identify interesting regions. MACAT also failed to evidence the MLL region (Figure 2 and Additional file 1, Figure S4).

#### Step 2

We completed the STEPath procedure by integrating chromosomal profile information in the module that performs gene set analysis.

The choice between gene (e.g., GSEA implemented in the limma R package [22] and sigPathway [23]) and sample permutations (e.g., Global test [21], Principal Coordinates and Hotelling's T^2 ^(PCOT2) [50] and Significance Analysis of Function and Expression (SAFE) [20]) has been an object of debate in the literature, as demonstrated by Efron's [26] and Goeman's [10] papers. After evaluating the limits and peculiarities of both permutational approaches, we chose the gene permutation model that seems to better fit our null hypothesis (as it is stated in step 2 - b of the algorithm description). We compared results obtained from STEPath with two other implementations of GSA both based on gene label permutations: GSEA implemented in the limma R package [22] and sigPathway [23]. STEPath is the only procedure that can identify the activated HOX gene set (Table 1). Dysregulation of HOX gene family members was found to be a dominant mechanism of leukaemic transformation induced by chimeric MLL oncogenes [51,52].

**Table 1:** Comparison of GSEA approaches

Rank	STEPath	STEPath - no correction
1	BioCarta;Erythropoietin mediated neuroprotection through NF-kB	BioCarta;Erythropoietin mediated neuroprotection through NF-kB

2	SuperArray;Homeobox (HOX) Genes	BioCarta;Induction of apoptosis through DR3 and DR4/5 Death Receptors

3	BioCarta;The IGF-1 Receptor and Longevity	BioCarta;Roles of -arrestin-dependent Recruitment of Src Kinases in GPCR Signaling

4	BioCarta;Induction of apoptosis through DR3 and DR4/5 Death Receptors	SuperArray;Homeobox (HOX) Genes

5	BioCarta;IL12 and Stat4 Dependent Signaling Pathway in Th1 Development	BioCarta;HIV-I Nef negative effector of Fas and TNF

6	BioCarta;HIV-I Nef negative effector of Fas and TNF	TCA Cycle;Metabolic Process

7	BioCarta;Roles of -arrestin-dependent Recruitment of Src Kinases in GPCR Signaling	hsa00310;Lysine degradation

8	TCA Cycle;Metabolic Process	hsa03018;RNA degradation

9	hsa00310;Lysine degradation	hsa05014;Amyotrophic lateral sclerosis (ALS)

10	hsa03018;RNA degradation	SuperArray;Stress/Toxicity PathwayFinder

**Rank**	**GSEA - limma**	**sigPathway**

1	B Cell Receptor Signaling Pathway;Cellular Process	BioCarta;Caspase Cascade in Apoptosis

2	hsa03018;RNA degradation	KEGG:03050;Proteasome

3	SuperArray;G-Proteins/Signaling Molecules	KEGG:04130;SNARE interactions in vesicular transport

4	TNF-alpha/NF-kB Signaling Pathway;Cellular Process	SuperArray;Heat Shock Proteins

5	hsa00510;N-Glycan biosynthesis	Proteasome Degradation;Physiological Process

6	hsa04142;Lysosome	KEGG:00380;Tryptophan metabolism

7	SuperArray;Autophagy	BioCarta;FAS signaling pathway (CD95)

8	BioCarta;Erk and PI-3 Kinase Are Necessary for Collagen Binding in Corneal Epithelia	KEGG:04612;Antigen processing and presentation

9	Translation Factors;Cellular Process	KEGG:03020;RNA polymerase

10	BioCyc;glyoxylate cycle II	KEGG:00020;Citrate cycle (TCA cycle)

Rank comparison of tested GSA for the most 10 up-regulated gene sets. STEPath is the only procedure that was able to identify the activated HOX gene set with a best rank using the corrected expression value based on chromosome profile.

To evaluate the contribution of chromosome profile information, we performed our GSA procedure (STEPath) with and without chromosome profile correction. In both cases, STEPath was able to identify the HOX gene set probably due to a separate evaluation of up- and down-regulated genes. However, the integration of gene location with gene set analysis allows the combination of different levels of biological information (co-expressed/regulated genes) and helps to correctly identify disease-related genes, since they have a different position in a rank evaluation (Table 1). To confirm this effect, we also ran limma GSEA using the chromosome profile correction. 55.5% of the common gene sets resulted with lower q-values when the analysis was integrated by the correction for chromosome profile. Furthermore, this correction was able to filter out the glyoxylate cycle, present in bacteria, fungi, yeast and plants (Table 2). This demonstrates that the correction enhances the discovery of disease-related genes, also filtering for apparently not informative pathways (in this case because specific for bacteria, fungi, yeast and plants).

**Table 2:** GSEA approach results running limma GSEA with and without chromosome profile correction

Rank	GSEA - limma	q-value	GSEA - limma - corrected	q-value
1	B Cell Receptor Signaling Pathway;Cellular Process	1.62E-05	B Cell Receptor Signaling Pathway;Cellular Process	2.68E-05

2	hsa03018;RNA degradation	1.44E-03	SuperArray;G-Proteins/Signaling Molecules	1.33E-03

3	SuperArray;G-Proteins/Signaling Molecules	1.53E-03	hsa03018;RNA degradation	2.31E-03

4	TNF-alpha/NF-kB Signaling Pathway;Cellular Process	3.79E-03	TNF-alpha/NF-kB Signaling Pathway;Cellular Process	2.93E-03

5	hsa00510;N-Glycan biosynthesis	4.20E-03	Translation Factors;Cellular Process	5.30E-03

6	hsa04142;Lysosome	8.78E-03	hsa00510;N-Glycan biosynthesis	5.40E-03

7	SuperArray;Autophagy	8.95E-03	hsa04142;Lysosome	6.40E-03

8	BioCarta;Erk and PI-3 Kinase Are Necessary for Collagen Binding in Corneal Epithelia	9.36E-03	SuperArray;Autophagy	8.21E-03

9	Translation Factors;Cellular Process	9.38E-03	hsa05110;Vibrio cholerae infection	9.42E-03

10	BioCyc;glyoxylate cycle II	1.06E-02	BioCarta;Erk and PI-3 Kinase Are Necessary for Collagen Binding in Corneal Epithelia	9.42E-03

Limma GSEA algorithm was run using the chromosome profile correction. Significance of the differentially expressed gene sets increases in comparison with results obtained without introducing chromosome profile correction, suggesting that it targets disease-related genes.

### Meta-analysis of LGMDs

We applied STEPath in a meta-analysis approach involving expression datasets of limb girdle muscular dystrophies type 2A (LGMD2A, calpainopathy), type 2B (LGMD2B, dysferlinopathy) and type 2I (LGMD2I). We built a meta-dataset combining LGMD2A from two distinct datasets. The first was published by Bakay [53] (GEO series ID: GSE3307); the second dataset was published by Sáenz [54] (GEO series ID: GSE11681). Meta-dataset details are listed in Table 3. Downloaded CEL files were processed using gene-based custom CDF [43]. We retrieved expression for 11,302 distinct genes. Following a visual inspection of the quantiles distribution (boxplot), we excluded 7 control samples from dataset GSE3307 (see Additional file 1; Figure S7). Gene expression data were then globally normalized using the RMA procedure [45].

**Table 3:** Details of muscle disease dataset.

Disease	Number of samples	Case study	Series ID	Platform	Description
LGMD2A	10	L/S	GSE3307	HGU133A	MUSCULAR DYSTROPHY, LIMB-GIRDLE, TYPE 2A (calpainopathy)

nLGMD2A	10	L/S	GSE11681	HGU133A	MUSCULAR DYSTROPHY, LIMB-GIRDLE, TYPE 2A (calpainopathy)

LGMD2B	10	L/S	GSE3307	HGU133A	MUSCULAR DYSTROPHY, LIMB-GIRDLE, TYPE 2B (Dysferlinopathy, Miyoshi distal myopathy)

LGMD2I	7	L/S	GSE3307	HGU133A	MUSCULAR DYSTROPHY, LIMB-GIRDLE, TYPE 2I

BMD	5	S	GSE3307	HGU133A	MUSCULAR DYSTROPHY, BECKER TYPE

DMD	10	S	GSE3307	HGU133A	MUSCULAR DYSTROPHY, PSEUDOHYPERTROPHIC PROGRESSIVE, DUCHENNE TYPE

FSHD	14	S	GSE3307	HGU133A	MUSCULAR DYSTROPHY, FACIOSCAPULOHUMERAL

AQM	5	S	GSE3307	HGU133A	ACUTE QUADRIPLEGIC MYOPATHY

SPG4	4	S	GSE3307	HGU133A	SPASTIC PARAPLEGIA 4, AUTOSOMAL DOMINANT

ALS	9	S	GSE3307	HGU133A	AMYOTROPHIC LATERAL SCLEROSIS 1

X_EDMD	4	S	GSE3307	HGU133A	EMERY-DREIFUSS MUSCULAR DYSTROPHY, 1 (X-linked)

AD_EDMD	4	S	GSE3307	HGU133A	EMERY-DREIFUSS MUSCULAR DYSTROPHY, AUTOSOMAL DOMINANT

AbNORM	11	L/S	GSE3307	HGU133A	NORMAL

Ctrl	10	L/S	GSE11681	HGU133A	NORMAL

General information about muscular disease meta-dataset. S: Skeletal Muscular disease dataset; L: LGMD analysis.

**Figure 3:**
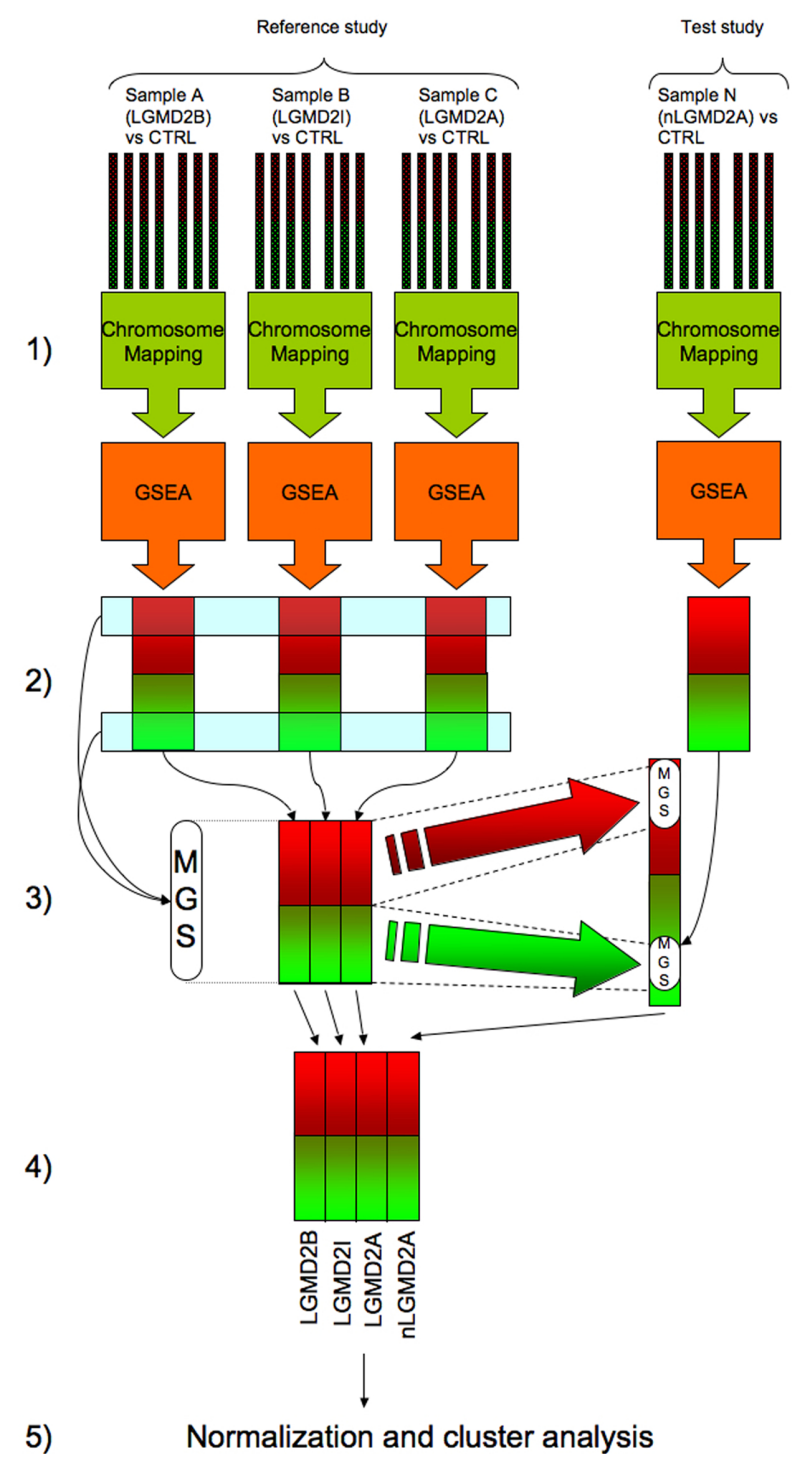
**LGMDs analysis workflow cartoon**. 1) Independent application of STEPath to the N considered datasets (e.g., for analysis of LGMDs LGMD2A, LGMD2B, LGMD2I from GSE3307 and nLGMD2A from GSE11681). 2) Selection of the Main Gene set Signature (MGS) from GSE3307 dataset. Selection was performed by identifying gene sets having an expression value upper or lower (for up- or down-regulated regions, respectively) than average of expression of all significant gene sets. 3) Extraction of the MGS expression values from all datasets considered. 4) Matrix construction. 5) Normalization and cluster analysis.

We applied 4 individual STEPath procedures to CTRLs vs LGMD2A (GSE3307), CTRLs vs LGMD2A (GSE11681), CTRLs vs LGMD2B (GSE3307) and CTRLs vs LGMD2I (GSE3307), where CTRLs are normal muscle controls from healthy donors (Ctrl plus AbNORM in Table 3). Ensembl features with no corresponding EntrezGene IDs as well as features/genes belonging to the Y chromosome were filtered out. We used STEPath scores to perform gene set meta-analysis (Step 3) (Figure 3).

In general, methods for meta-analysis were based on the union or the intersection of lists of differentially expressed genes derived from multiple studies of the same biological issue. However, these lists have little overlap because of biological and technical variability [55,56], while pathway analysis often generates improved consistency [16]. An explanation for the reduced discrepancies in the results of the microarray data based on biological gene sets analysis, compared with the over imposition of the groups of differentially expressed genes derived from different studies, may be the correlation of differentially expressed genes. In fact, the differences in their relative expression may be so small that the choice of top-ranked genes is highly dependent on the studies or analysis method from which genes are inferred, as reported in [16]. Moreover there is the possibility that gene sets with no differentially expressed genes (due by the choice of the threshold) will show an aberrant global expression pattern because most of the genes in the set have an even small, but coordinated change (up- or down-regulation) allowing their identification in different studies and increasing their comparability.

Recently, Shen [11] proposed the integrated Meta-Analysis of Pathway Enrichment approach (MAPE_I), combining statistical significance at the gene and pathway level based on a gene-wise and sample-wise permutation test. Our framework is focused on this aspect of meta-analysis, but it introduces the possibility to compare different conditions, highlighting peculiarities of each one.

To produce the main gene set signature (MGS, the union of the pathology signature; see Methods), we chose GSE3307 (LGMD2A, LGMD2B and LGMD2I) as a reference study. The signature was composed of 70 gene sets: 55 Gene Ontology and 15 biological pathways that were used to build the gene set matrix (see Methods).

**Figure 4:**
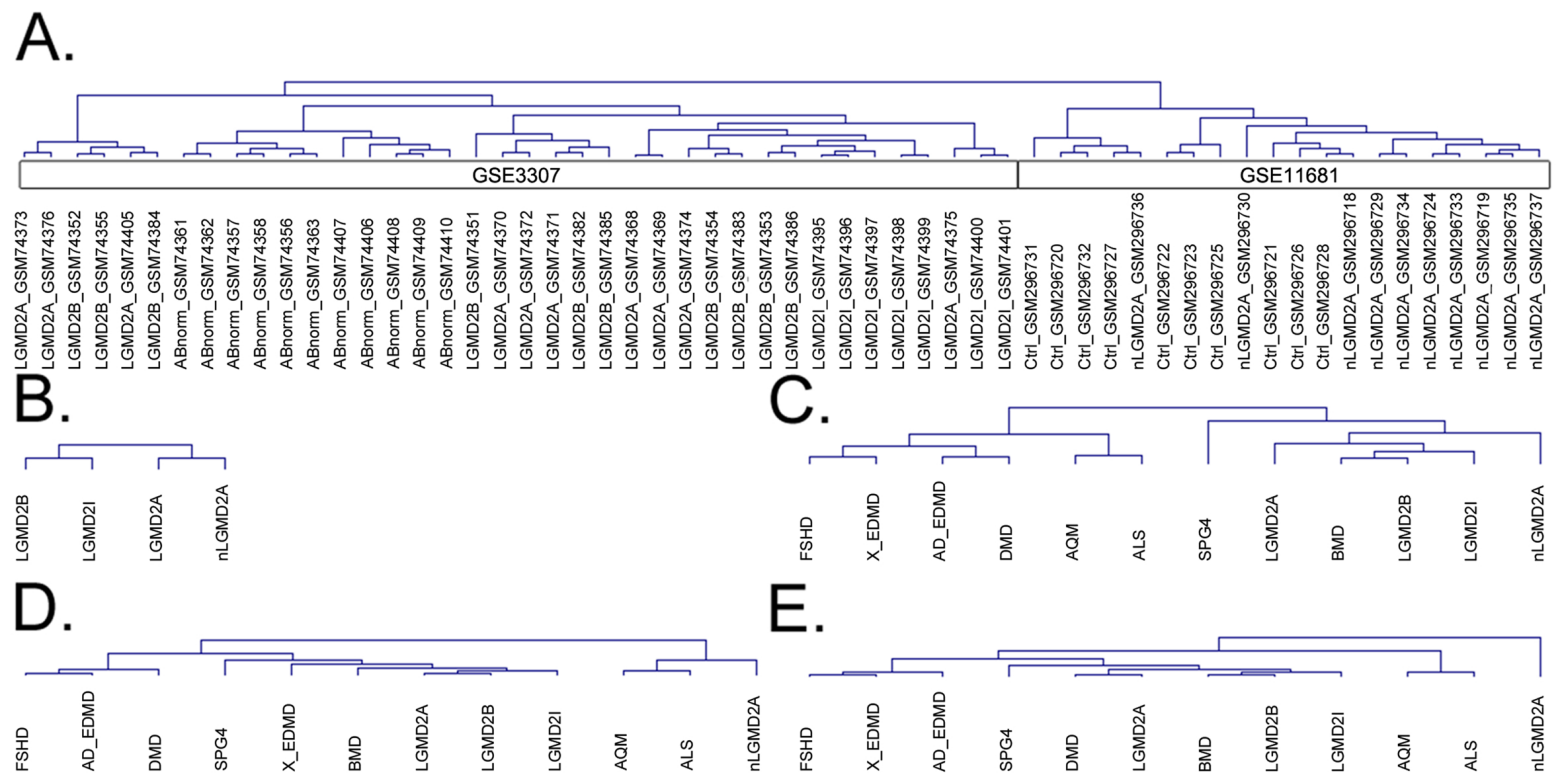
**Cluster analysis for LGMD expression meta-dataset**. A. Unsupervised cluster analyses on gene expression data from meta-dataset of LGMDs. Samples of each dataset are grouped separately: the GSE3307 dataset on the left branch and the GSE11681 dataset on the right. B. Cluster tree of unsupervised cluster analysis of the Main Gene set Signature matrix from LGMDs dataset: segregation is guided by disease type and not by dataset. C. Unsupervised cluster analysis result of gene set scores calculated with the STEPath algorithm. LGMD2A from two different datasets clustered together. D. Unsupervised cluster analysis performed on gene set scores calculated with sigPathway algorithm (NTk was the score used, defined as the gene-based normalized statistic obtained by permuting genes). E. Unsupervised cluster analysis based on gene set scores calculated with the GSEA limma algorithm. Clustering based on gene set scores calculated with both sigPathway and limma algorithms failed to link together the two LGMD2A different datasets.

Cluster analysis of the gene set matrix (derived from the main signature) shows that the two LGMD2As from different datasets are linked, and LGMD2B and LGMD2I segregate separately (Figure 4B; for data matrix see Additional file 3).

We then performed unsupervised cluster analysis [57] on gene expression data. This analysis highlighted two main branches that separate GSE3307 and GSE11681 datasets (Figure 4A). This demonstrates that background noise and the presence of a batch effect is strong enough to overcome disease-specific signals at gene expression levels (Figure 4A). Moreover, cluster analysis failed to separate pathologies (see Additional file 1; Figure S8) using significant differentially expressed genes only (significant genes identified by SAM with False Discovery Rate = 0).

Our approach overcomes the main limitations of gene expression meta-analysis and demonstrates that it is useful to reveal gene set signatures that discriminate different pathologies. In this way, we can evaluate the main signature discrimination/association power, projecting it into the second study (Figure 3, point 3).

### Meta-analysis of Skeletal Muscular diseases

We extended the analysis performed for LGMDs including more variability with different skeletal muscular diseases (all samples reported in the Table 3).

Raw expression files (CEL files) were downloaded from the GEO database [7] (GEO series IDs GSE3307 and GSE11681) and processed using a gene-based custom CDF, as previously discussed. Normalized gene expression for 11,302 genes was used in the STEPath analysis. Extended datasets were used to evaluate if increased variability affects the meta-analysis procedure. We extracted signatures from all skeletal muscle diseases in the dataset GSE3307 to build the MGS used in the cluster analysis. Cluster analysis showed that increased variability of initial samples did not affect clustering results since LGMDs still clustered together; different datasets of LGMD2A were still in close proximity (Figure 4C; for matrix, see Additional file 4).

We compared STEPath meta-analysis results with a meta-analysis approach based on different GSA scores. Similarly for STEPath, we built a MGS matrix using scores derived by both sigPathway and GSEA (as implemented in limma). Clustering results of the MGS matrix from both sigPathway and GSEA failed to co-segregate the two LGMD2A datasets and the entire group of LGMDs (Figure 4D and 4E).

**Figure 5:**
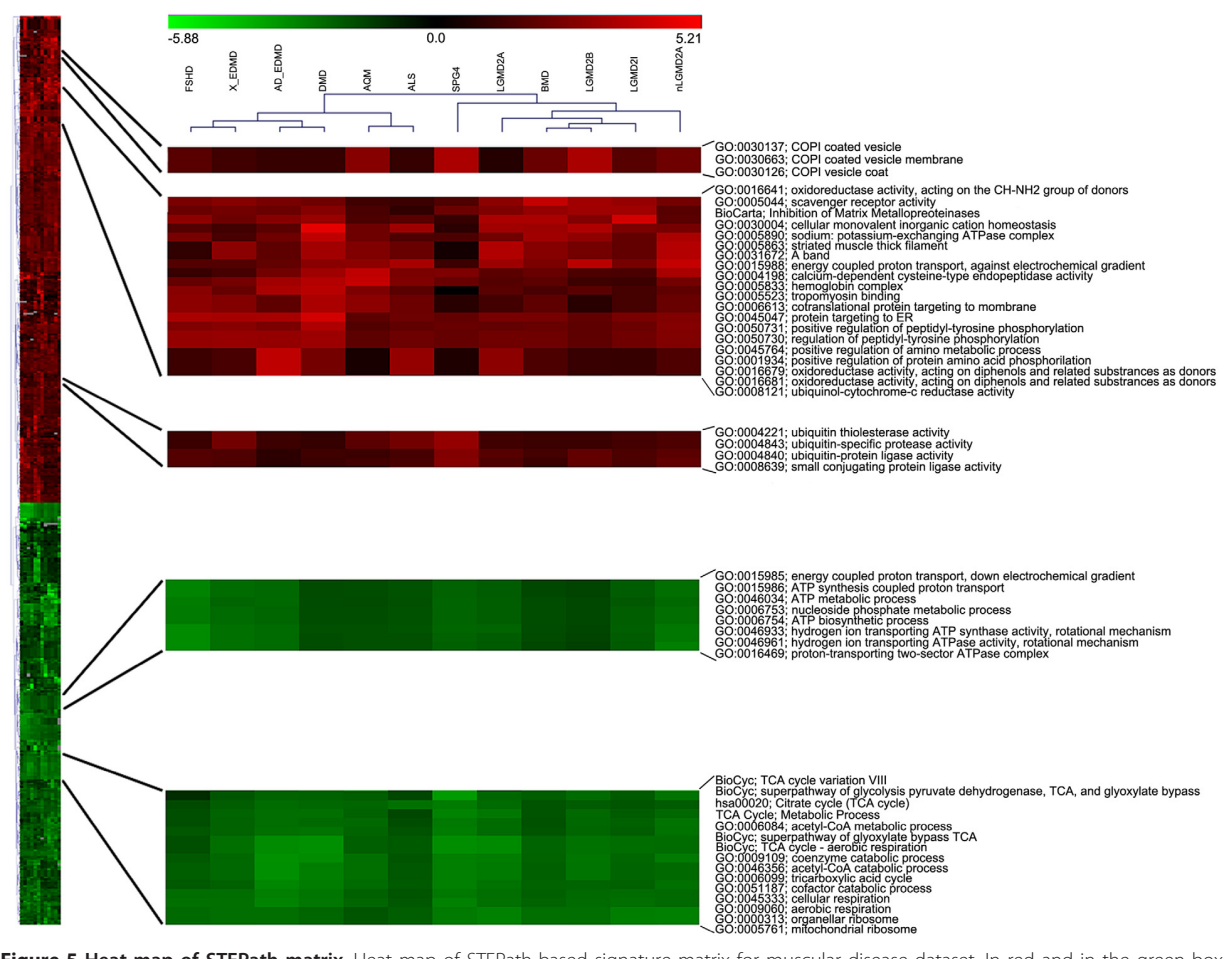
**Heat map of STEPath matrix**. Heat map of STEPath-based signature matrix for muscular disease dataset. In red and in the green box, up-regulated and down-regulated gene sets, respectively. Grey boxes are for gene sets that do not have up- or down-regulated elements.

Gene set clusters were analysed focusing on both shared and peculiar pathology responses. Down-regulated gene sets show several clusters with the same expression level. These gene sets mainly refer to aerobic respiration, the production of ATP and mitochondria (Figure 5). These results are in agreement with many microarray studies on skeletal muscle dystrophies [58] and a previous meta-analysis work that we performed to detect muscle atrophy signatures [59]. In many skeletal muscle pathologies, the rate of degradation of contractile proteins becomes greater than the rate of replacement, causing atrophy and modifying the balance requested for the maintenance of skeletal muscle mass. Ubiquitination function involved in protein degradation and gene sets for oxidative stress and mitochondrial function appear to be up-regulated, yet they are not discriminative among the pathologies. Gene sets involved in oxidoreductase activity (GO_MF: 0016641), scavenger receptor activity (GO_MF: 0005044) and regulation of amino acids (GO_BP: 0045764 and GO_BP: 0001934) are some examples (Figure 5). Recently, Kramerova [60] postulated that LGMD2A and other dystrophies (Duchenne Muscular Distrophy and Becker Muscular Dystrophy) are characterized by energy deficit and increased oxidative stress. We highlighted the activation of gene sets involved in antioxidant activity like GO_MF: 0016681, GO_MF: 0016679 and GO_MF:0016641 that referred to oxidoreductase activity.

Dysferlinopathy (LGMD2B) was characterized as dystrophies where dysferlin-deficient cells show abnormalities in vesicular trafficking [61]. LGMD2B also presents muscle inflammation with muscular monocytes and macrophages that show an increased phagocytic activity [62]. Efficient phagocytic activity depends on the presence of the coat protein complex type I (COPI) [63], a complex that plays an essential role in the trafficking of membrane vesicles. Our procedure reveals this relation between vesicle trafficking impairment and enhanced phagocytosis in LGMD2B as is demonstrated by a marked up-regulation of GO terms that refer to COPI-coated vesicles (GO_CC: 0030137, GO_CC: 0030663 and GO_CC: 0030126) (Figure 5).

**Figure 6:**
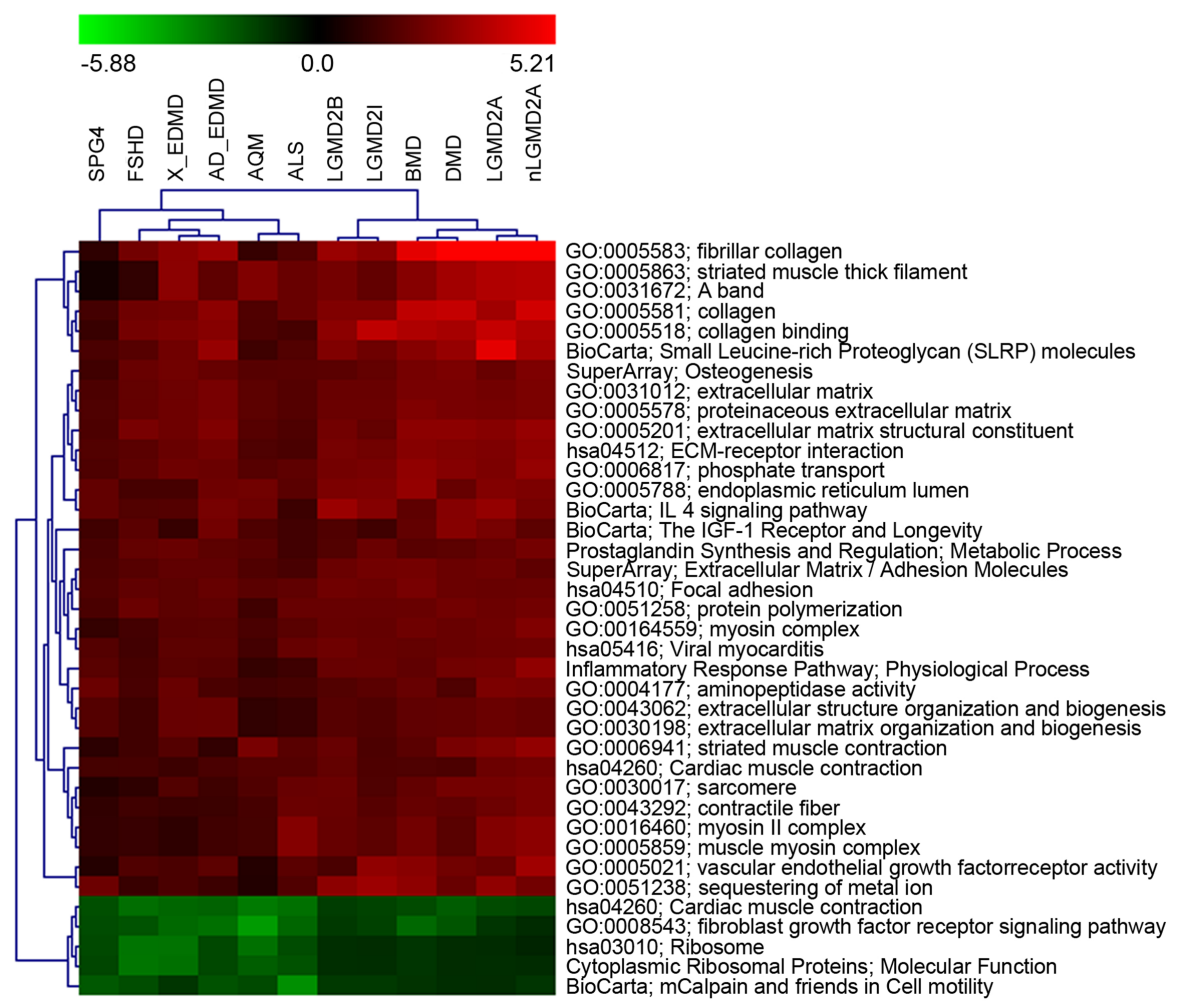
**Heat map of PTM analysis**. Heat map of PTM analysis searching for gene sets with marked up-regulation in LGMD2A pathologies with respect to other gene sets.

To search for discriminative gene sets among LGMD2A (Calpain 3; CAPN3 is the causative gene) and the other skeletal muscular diseases, we performed a template matching search [64]. In Figure 6 are reported hierarchical clusters of gene sets identified with p-value ≤ 0.05.

Recently, Beckman and Spencer [65] proposed that CAPN3 is involved in sarcomeric protein turnover and in the maintenance of sarcomere integrity. Collagen (GO_CC: 0005581), collagen binding (GO_MF: 0005518) and fibrillar collagen (GO_CC: 0005583) gene sets, involved in the maintenance of mechanical stability of muscle structure, sarcomere (GO_CC: 0030017), striated muscle thick filament (GO_CC: 0005863), A band (GO_CC: 0031672) and contractile fiber (GO_CC: 0043292) involved in the contraction process, appear to be up-regulated in LGMD2A (Figure 6). Gene sets previously described are also altered in LGMD2B patients that present mutations in the dysferlin gene (DYSF), which interacts with CAPN3. A secondary reduction of CAPN3 that can affect sarcomere structure stability in LGMD2B patients has also been demonstrated [66].

### Implementation

All functions to perform STEPath analysis are written in R (http://www.r-project.org/, version 2.10.1), and they are freely available as R package at http://gefu.cribi.unipd.it/papers/stepath under the AGPL3 licence. The implementation depends on bioconductor [67] version 2.5 (for affy R package [68]) and samr R package (http://CRAN.R-project.org/package=samr).

The present implementation is microarray platform-independent and potentially could be applied to any kind of gene-phenotype association score (SAM t-test, t-test).

## Conclusions

The algorithm we developed allows the analysis of gene expression data by integrating supplementary biological information to identify gene co-expression along the chromosomes and to perform a gene set analysis. The integration was initially tested on a leukaemia dataset, highlighting interesting imbalanced regions containing genes involved in ALL pathology: 1) MLL region on chromosome 11; 2) the region containing HOX gene cluster on chromosome 7; and 3) the region containing MEIS1 on chromosome 2. These regions are specifically enhanced by the STEPath algorithm and not by LAP or MACAT.

The second integration was tested on gene expression datasets both from leukaemia and skeletal muscle diseases evidencing the importance of integration of the chromosome profiles in the gene set analysis. Integrating two biological aspects in the STEPath algorithm (locus transcription that spills over into its physical neighbour loci and co-regulation of gene sets), we have demonstrated that STEPath produces gene set expression scores that are suitable to directly compare different diseases and studies to perform meta-analyses.

We applied STEPath and the meta-analysis approach to limb girdle muscular dystrophies (LGMDs), highlighting the co-segregation of two different studies of LGMD2A patients, and to a meta-dataset for inflammatory myopathies composed of both Affymetrix arrays and unpublished custom oligo arrays. Results of the study of inflammatory myopathies will be discussed in a separate paper.

## Methods

### STEPath algorithm

**Step 1**: The process to identify differentially expressed regions can be divided into four parts: I) computation of a per-gene statistic to measure differences in gene expression between two groups under investigation or in one group, if microarray experiments were performed in a competitive hybridization way, II) correction of the statistics based on the expression level of neighbourhood loci, III) identification of statistically differentially expressed regions by a permutational approach and IV) building of per-chromosome profile.

I) In this study, we used the SAM t-statistic to measure the association of genes to the phenotype of the two conditions. All SAM analyses were computed using two-class unpaired comparisons between a disease state versus a reference condition based on 100 permutations via the samr R package [69].

II) This step computes the local index of global activation (*Eup*) or inhibition (*Edown*) for every gene *i *considering the neighbour genes and the local gene density.

For any given gene *i*, *rup*_*i *_is the region centred in the transcription start site of the gene *i *(TSS*i*) that covers n = 2 up-regulated genes upstream TSS*i *and n = 2 up-regulated genes downstream TSS*i*. Given *rup*_*i*_, we can summarize the local gene expression contribution for up-regulated genes inside the region (*S'*_*i*_^*up*^), as described in equation 1:(1)

where *U *= {*u *∈ *rup*_*i*_|*S*_*u *_≥ 0}, and |*U*| denotes the cardinality of the set *U*.

We can also define the local gene density of up-regulated genes (ρ_up_) as reported in equation 2:(2)

*Eup*_*i *_is then calculated as define by equation (3):(3)

where *S'*^*up*^_*c *_is the mean of summarization values for all designed *rup *in the chromosome *c *and ρ^*up*^_*c *_is a per-chromosome estimation of the global up-regulated gene density. We defined per-chromosome global up-regulated gene density as the mean of all local densities for every up-regulated gene.

The ratio between S^'up^_i _and S^'up^_c _is meant to rescale regional expression compared to the average situation in the chromosome *c*, while the ratio between ρ^*up*^_*i *_and ρ^*up*^_*c *_is meant to favour regions presenting genes more densely distributed than the mean local densities of the chromosome.

In parallel, for any gene *i*, we defined *rdown*_*i *_and the summarization value *Edown*_*i *_as described for up-regulated genes. In this case, *U *is defined as follows:

III) We adopted a permutational approach to identify significantly different *Eup*_*i *_and *Edown*_*i*_. In particular, SAM statistics were randomly shuffled over gene positions. We applied procedure II) and III) to *B = 100 *of these permutations to compute *E'up*_*i,b *_and *E'down*_*i,b *_null distributions, where *1 ≤ b ≤ B *(null hypothesis *H*^*0*^: the region is not differentially expressed). We computed p-values for every window centred in gene *i *as the probability that *E'up*_*i *_or *E'down*_*i *_exceed respectively the observed *Eup*_*i *_and *Edown*_*i *_over *B *permutations. We then corrected p-values for multiple testing error using the Benjamini Hochberg FDR control (preprocessCore r package [70]).

IV) The final step was performed to produce a per-chromosome profile by scanning each chromosome gene by gene using a window of fixed length. For window size, we used the reference lengths for up- and down-windows defined as the average length of all *rup*_*i *_and *rdown*_*i *_for a given chromosome *c*. We used these dimensions because they are in accordance with the clustering scale dimensions found in mammalian genomes by Firneisz [71] and Farr [72] (see Additional file 2, Table S5). We count significant (q-value < = 0.05) up- or down-regulated window expression values present in the fixed windows that slide gene by gene. The up and down profile was built respectively as the fraction of significant *Eup *or *Edown *present in the sliding windows.

### Statistics smoothing based on chromosome profile function

SAM statistic *S *was corrected according to equation 4:(4)

where *SS*_*i *_is the smoothed statistic value for gene *i*, and *pf*_*i *_is the profile value in the chromosome region identified by the gene *i *(see step IV of the previous paragraph).

**Step 2**: the GSA module can be divided into two parts: 1) computation of per-gene set scores and 2) identification of significant gene sets.

1) We implemented the measurement of gene set scores as the mean of the corrected SAM statistics, *SS*. In particular, we performed the GSA computing up- and down-regulated gene contributions separately. Let the indices *k*, *k = 1,...,K *denotes the *k*^*th *^gene set, and *i, i = 1,..,I *denotes the *i*^*th *^gene. We defined an incidence matrix *M *with dimensions *K *× *I*, where *M*_*k,i *_*= 1 *denotes the presence of gene *i *in the *k*^*th *^gene set, and *M*_*k,i *_*= 0 *denotes the absence of gene *i *in the *k*^*th *^gene set. We computed a gene set up-regulation value *Gup*_*k *_for the *k*^*th *^gene set as the mean of *SS*_*i∈k *_*≥ 0 *and similarly for down-regulated values (*Gdown*_*k*_).

2) To assess the significance of gene sets, we adopted a gene-based permutational scheme to compute null distributions of *SS*: *SS'*_*b*_. We applied *B = 100 *permutations on *S*, and for each permutation *b*, we smoothed *S'*_*b *_to compute *SS'*_*b *_(null hypothesis *H*^*0*^: the gene set shows the same pattern of association with the phenotype compared to the rest of genes). We applied the procedure described in 1) to compute *Gup'*_*b *_and *Gdown'*_*b *_null distributions for each gene set. P-values were then calculated for *Gup*_*k *_and *Gdown*_*k *_independently as the probability that *Gup'*_*k *_or *Gdown'*_*k *_exceed the observed gene set score *Gup*_*k *_or *Gdown*_*k *_over *B = 100 *permutations. P-values were finally corrected according to Benjamini Hochberg FDR control.

**Step 3**: step 3 compares different pathologies and different studies. The starting points are *Gup *and *Gdown *summarization values of the gene sets from step 2.

A reference study was defined as the one with greater variability. From each pathology in the reference study, a gene set signature was extracted. It was defined as the significant gene sets (q-value < = 0.05) with either *Gup *or *Gdown *exceeding the mean score of significant gene sets.

We called the union of the pathology signatures main gene set signature (MGS). Using the MGS, corresponding summarization values from all the pathologies in all studies (from the reference study and from the validating one) were extracted and a matrix (MGS matrix) was produced, where columns were the different pathologies, and rows were summarization values for every gene set on the signature. To make comparable values of each gene set signature among different pathologies, quantile normalization was applied. The normalized matrix provides a direct comparison of gene set activation and inhibition across pathologies and studies. We adopted this strategy because signatures identified in one study should be identified in independent studies for the same pathology; related pathologies from different studies should cluster together.

### Custom CDF

We developed a gene-based custom Chip Definition File (CDF) by re-mapping probes of Affymetrix HGU133plus2 and HGU133A chips on the ENSEMBL gene database (ver 56). Gene-based custom CDFs were generated as follows: i) matching of ensemble gene sequences with all probes present in a given gene chip (HGU133plus2, HGU133A); ii) filtering out of non-specific probes (probes that match more than one gene sequence); iii) grouping of remaining probes in meta-probe sets with at least 4 members; iv) discarding all probes not belonging to any meta-probe set defined in point iii) [43]. None of the identified genes share TSS in both of the designed CDFs. We adopted this strategy because gene sets are defined as groups of genes and not as groups of transcripts that could derive from alternative TSS of the same gene.

### Gene sets

We compiled a collection of gene sets using various public databases. We used gene sets from Gene Ontology (GO) (6,466 gene sets derived from Biological Process, Molecular Function and Cellular Component), 204 KEGG pathways, 161 ByoCyc pathways, 102 Superarray pathways and 111 wiki pathways. Only gene sets with members in the CDF were used in the analysis.

### Normalization

Multichip normalization was performed using RMA as implemented in the affy bioconductor package.

The gene set expression matrix was normalized by quantile normalization as implemented in R package preprocessCore.

### Cluster and Template Matching Analysis

All cluster analyses were performed using the Euclidean distance with complete linkage method. We used the Hierarchical Cluster Analysis (HCL) implemented in the TMeV suite (version 3.1) [73].

PTM analysis was performed using the PTM function implemented in the TMeV suite and setting p-value threshold at 0.05.

### R packages

Limma GSEA was performed using the limma bioconductor R package.

SigPathway analysis was performed using the sigpathway bioconductor R package.

MACAT analysis was performed using the macat bioconductor R package.

## List of abbreviations

LAP: Locally Adaptive statistical Procedure; MACAT: MicroArray Chromosome Analysis Tool; AML: acute myeloid Leukaemia; ALL: acute lymphoblastic leukaemia; GSA: Gene Set Analysis; SAM: Statistical Analysis of Microarray; KEGG: Kyoto Encyclopedia of Genes and Genomes; GEO: Gene Expression Omnibus; RMA: Robust Multiarray Averaging; PAM: Predicted Analysis of Microarray; PCOT2: Principal Coordinates and Hotelling's T^2^; SAFE: Significance Analysis of Function and Expression, LGMDs: Limb Girdle Muscular Dystrophies; CTRL: Control; MAPE, Meta-Analysis of Pathway Enrichment; MGS: Main Gene set Signature; ATP: Adenosine triphosphate; GO_BP: Gene Ontology Biological Process; GO_MF: Gene Ontology Molecular Function; GO_CC: Gene Ontology Cellular Component; AGPL3: GNU Affero General Public License; FDR: False Discovery Rate; CDF: Chip Definition File; HCL: Hierarchical Cluster Analysis; TMeV: TIGR MultiExperiment Viewer; PTM: Pavlidis Template Matching.

## Competing interests

The authors declare that they have no competing interests.

## Authors' contributions

PM implemented the STEPath algorithm, performed all of the statistical and bioinformatic analyses. DR, GS and CR participated in the design of the study, revised the manuscript and participated in the investigation of the significant gene sets and interpretation of the results. GL and SC conceived and supervised the study, wrote the manuscript, coordinated the work and the interpretation of the results. All authors read and approved the final version of the manuscript declaring that they have no potential conflicts of interests.

## Supplementary Material

Additional file 1 **Additional figures**. Word document containing supplementary figures. The figures are provided one per page with a short description. Click here for file

Additional file 2 **Additional tables**. Excel spreadsheet file containing supplementary tables. External link to NCBI and Gene Card databases are provided for genes explored in the chromosome regions described in the text. Click here for file

Additional file 3 **LGMDs dataset data matrix**. Text file containing data matrix for LGMD dataset gene set meta-analysis. Click here for file

Additional file 4 **Muscle disease dataset data matrix**. Text file containing data matrix for muscular disease dataset gene set meta-analysis. Click here for file
