# Supplementary material for: Statistical Test of Expression Pattern (STEPath): a new strategy to integrate gene expression data with genomic information in individual and meta-analysis studies
Source: BMC Bioinformatics. 2011 Apr 11;12:92. doi: 10.1186/1471-2105-12-92 (PMC3094239; doi:10.1186/1471-2105-12-92)
Supplement: Additional file 1 — Additional figures. Word document containing supplementary figures. The figures are provided one per page with a short description. [file 1471-2105-12-92-S1.DOC]

**Additional file 1**


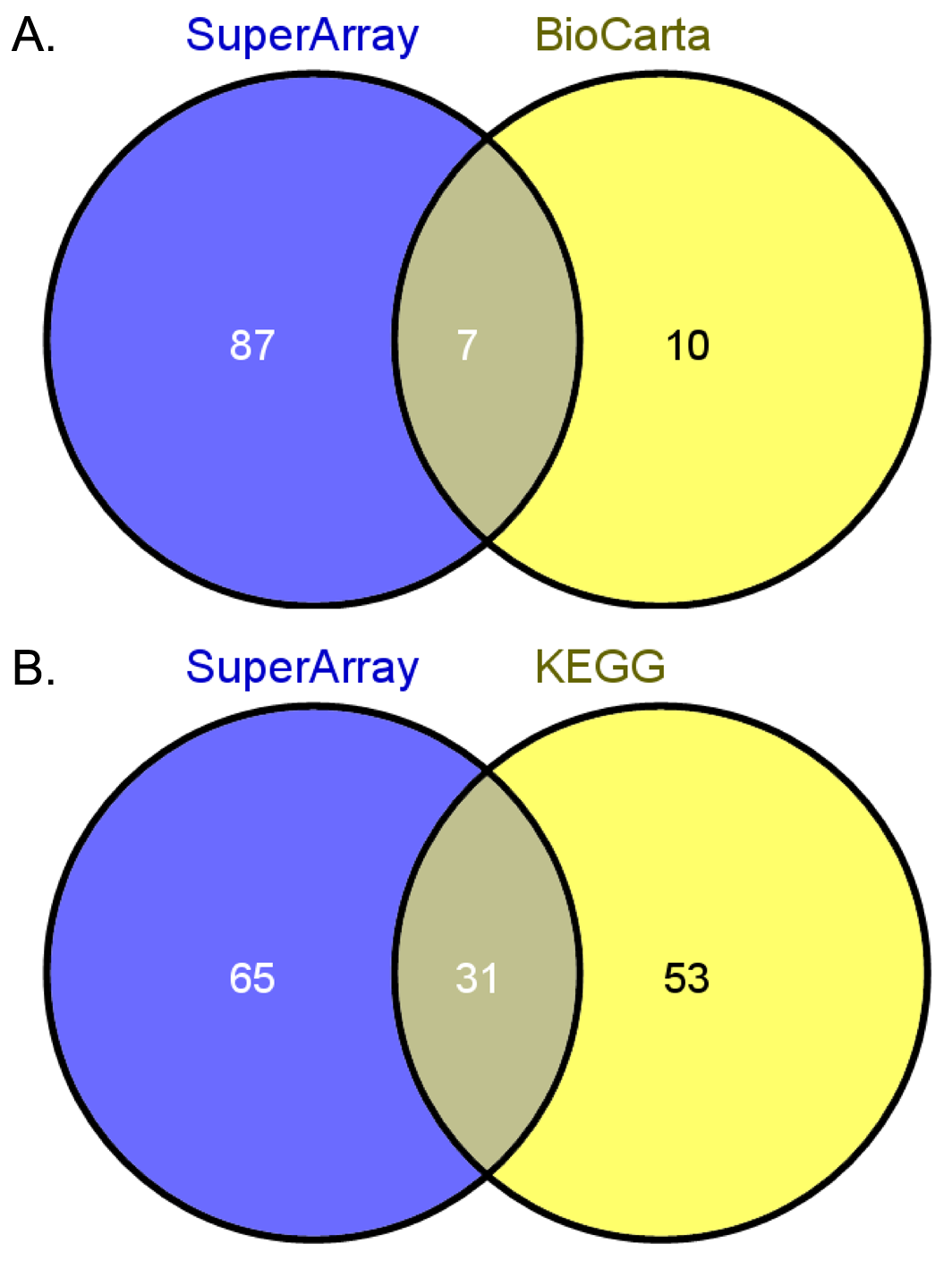


**Figure S1.** A) Venn diagram showing overlapping between Interferon Type I as annotated in SuperArray (blue circle) and BioCarta (yellow circle). The BioCarta gene set for IFN type I counts 17 members while 94 members are present in SuperArray gene set. B) Venn diagram showing overlapping between pathways for Extracellualr Matrix components (ECM) as annotated in SuperArray (blue circle) and KEGG (yellow circle). ECM pathway counts 84 members in KEGG and 96 in SuperArray. Differences in the composition of gene sets that describe the same process could denote different aspects of gene set knowledge.


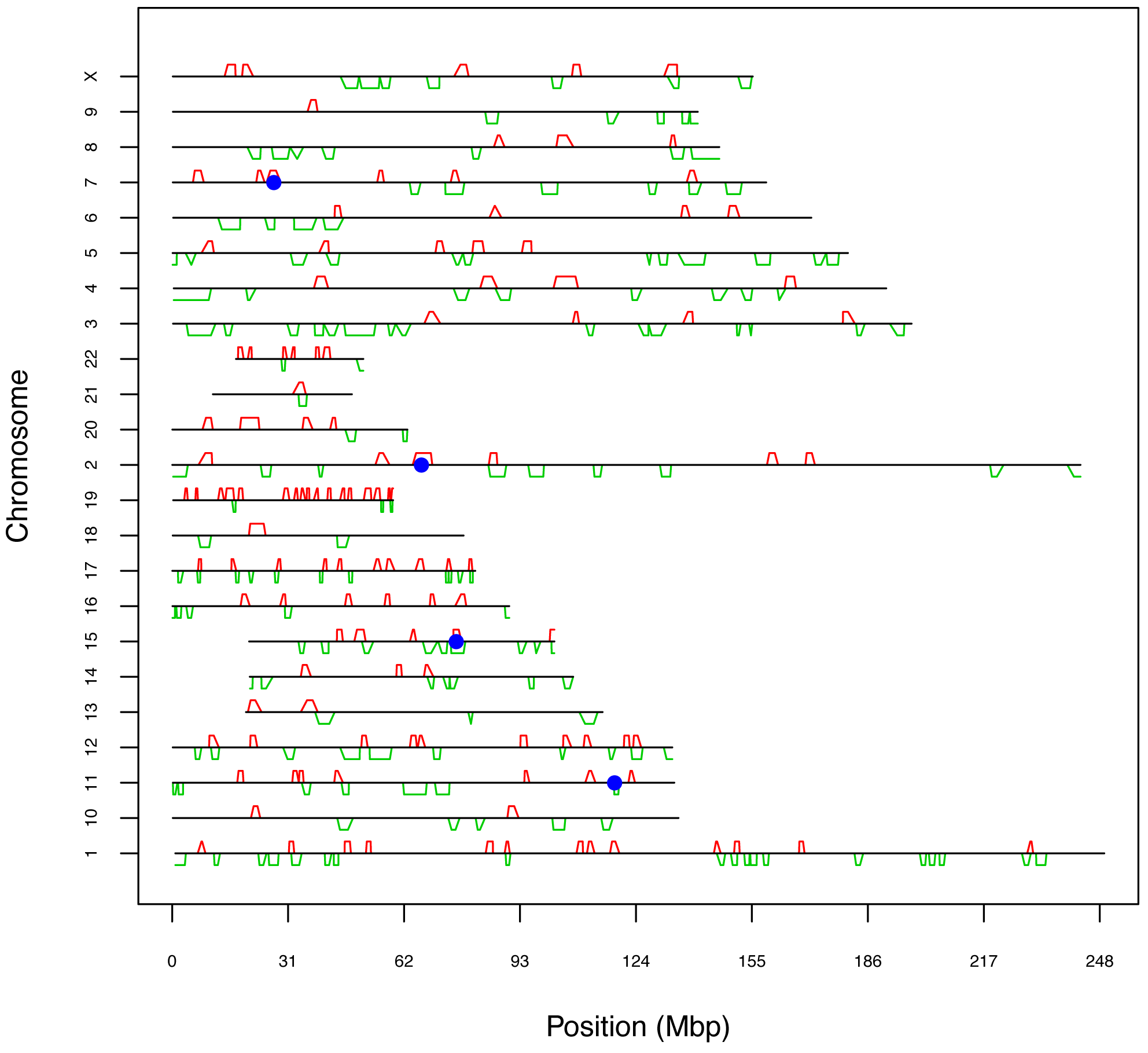


**Figure S2.** Graphical representation of putative imbalanced regions identified with the chromosome mapping module implemented in STEPath. Data refer to leukaemia dataset. Red and green curves represent up- and down-regulated imbalanced regions respectively. Blue dot are used to indicate HOX cluster on chromosome 7, the region containing Meis homeobox 1 (MEIS1) on chromosome 2, the chondroitin sulfate proteoglycan 4 (CSPG4/NG2) on chromosome 15 and MLL region on chromosome 11. Chromosomes were numbered in Y axis (1 – 22 plus X chromosome because genes referring to chromosome Y were filtered out because paucity of gene expression data preclude chromosome mapping application) while gene position was described in Mega base pairs (Mbp) in X axis.


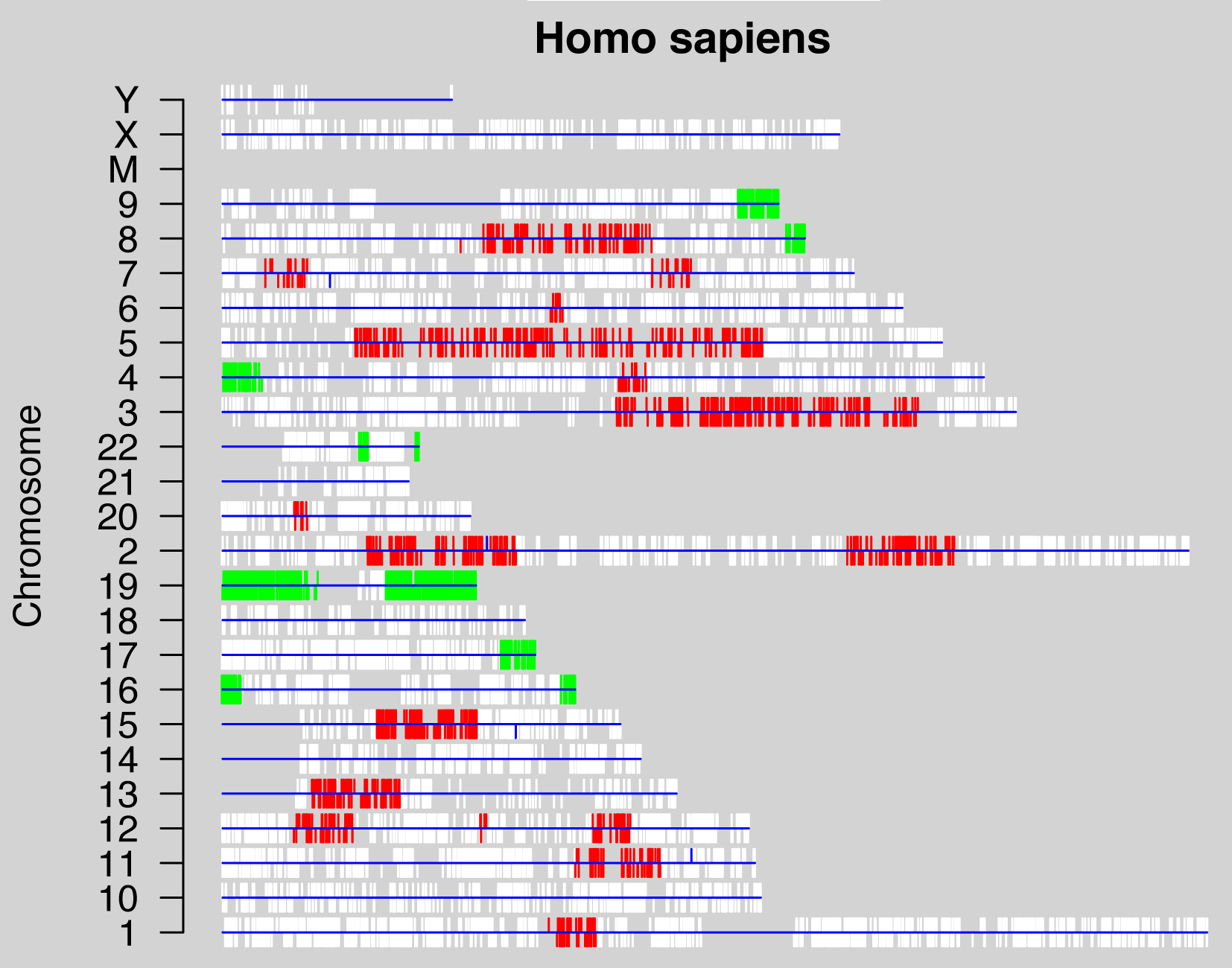


**Figure S3.** Graphical representation of putative imbalanced region identified with LAP algorithm performing analysis with suggested permutations (10,000). In red and green are represented up- and down-regulated chromosome regions respectively. Blue vertical lines are used to indicate HOX cluster on chromosome 7, the region containing Meis homeobox 1 (MEIS1) on chromosome 2, the chondroitin sulfate proteoglycan 4 (CSPG4/NG2) on chromosome 15 and MLL region on chromosome 11. Only the region associated with MEIS1 appears to be imbalanced. Data refer to leukaemia dataset.


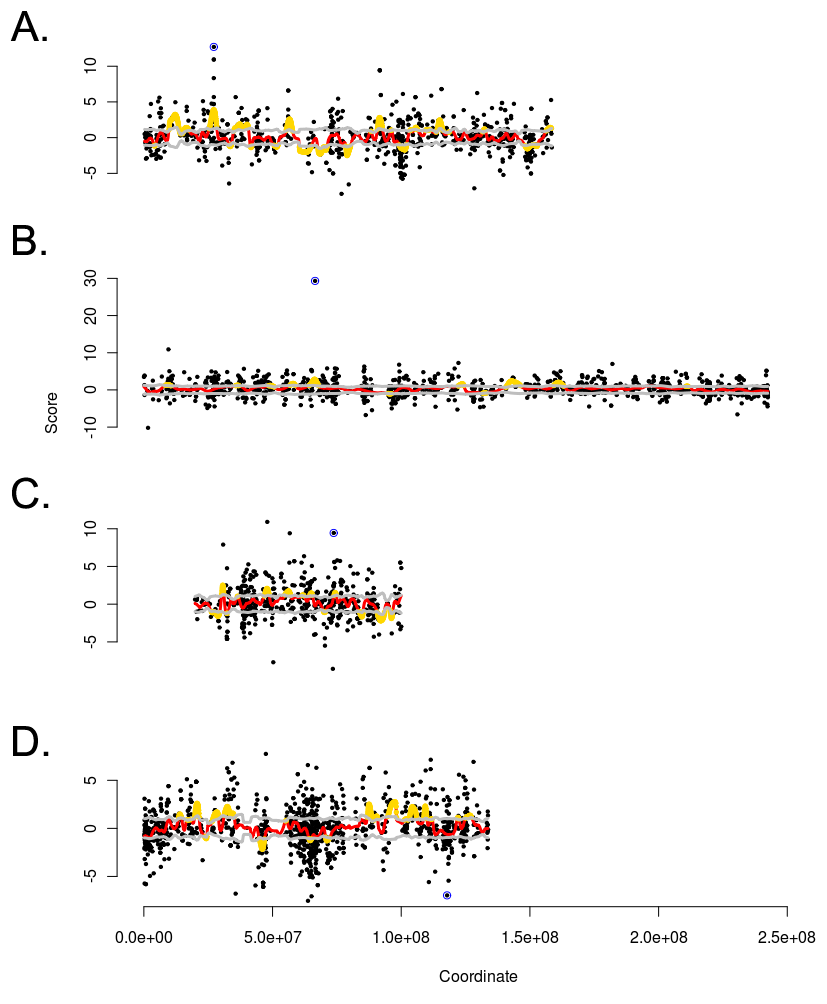


**Figure S4.** Graphical representation of imbalanced regions identified with MACAT in chromosome 7 (A), chromosome 2 (B), chromosome 15 (C) and chromosome 11 (D). Analyses were performed using suggested number of permutation (1,000). Red line describes chromosome profile that change color (yellow) in regions statistically imbalanced. Grey lines describe the sliding average of the 0.025 and 0.975 quantiles of the permuted scores. Blue dot are used to indicate HOX cluster on chromosome 7, the region containing Meis homeobox 1 (MEIS1) on chromosome 2, the chondroitin sulfate proteoglycan 4 (CSPG4/NG2) on chromosome 15 and MLL region on chromosome 11. MLL region was not detected. Data refer to leukaemia dataset.


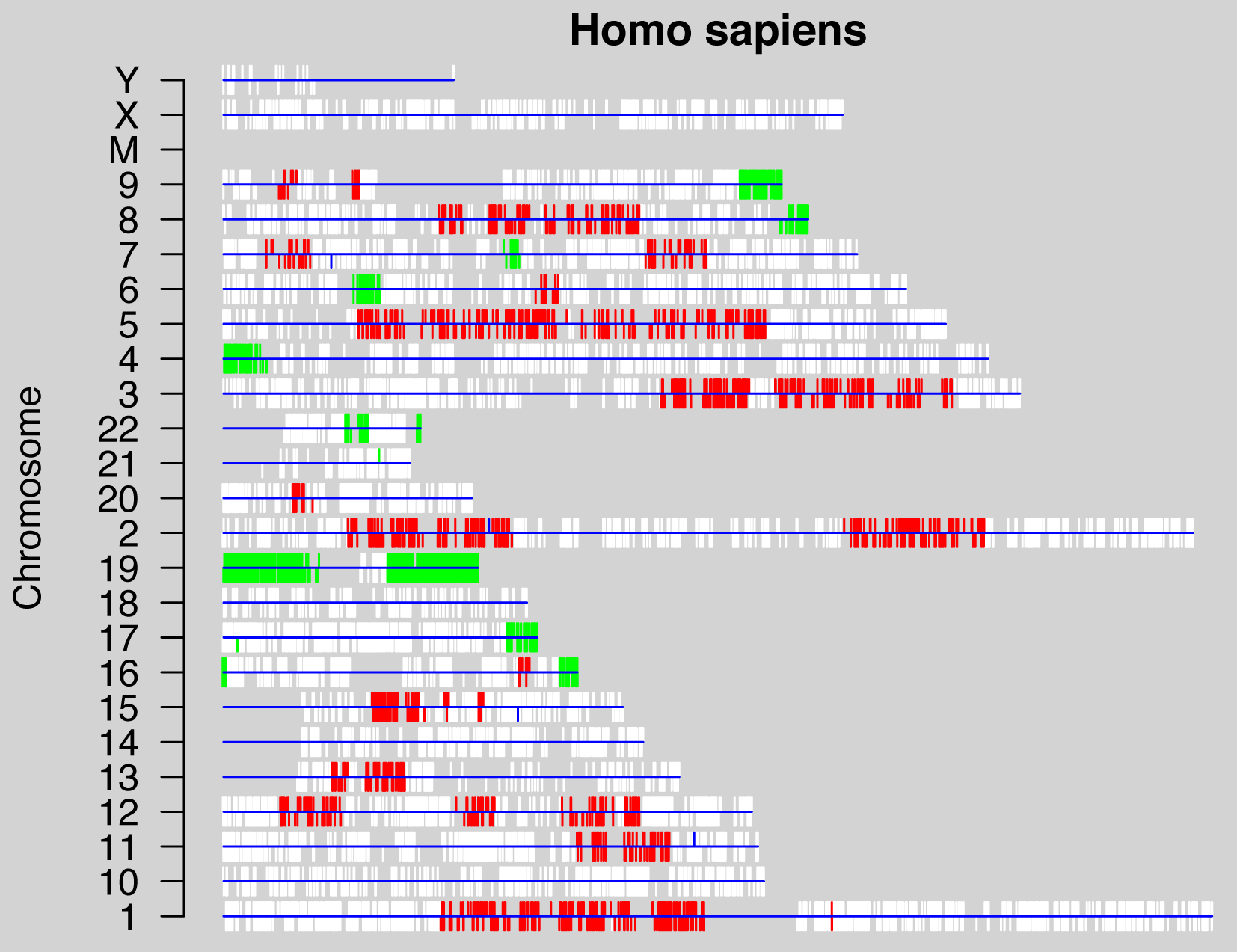


**Figure S5.** Graphical representation of putative imbalanced regions identified using LAP algorithm with 100 permutations. In red and green are represented up- and down-regulated chromosome regions respectively. Blue vertical lines are used to indicate HOX cluster on chromosome 7, the region containing Meis homeobox 1 (MEIS1) on chromosome 2, the chondroitin sulfate proteoglycan 4 (CSPG4/NG2) on chromosome 15 and MLL region on chromosome 11. Only the region associated with MEIS1 appears to be imbalanced. Data refer to leukaemia dataset.


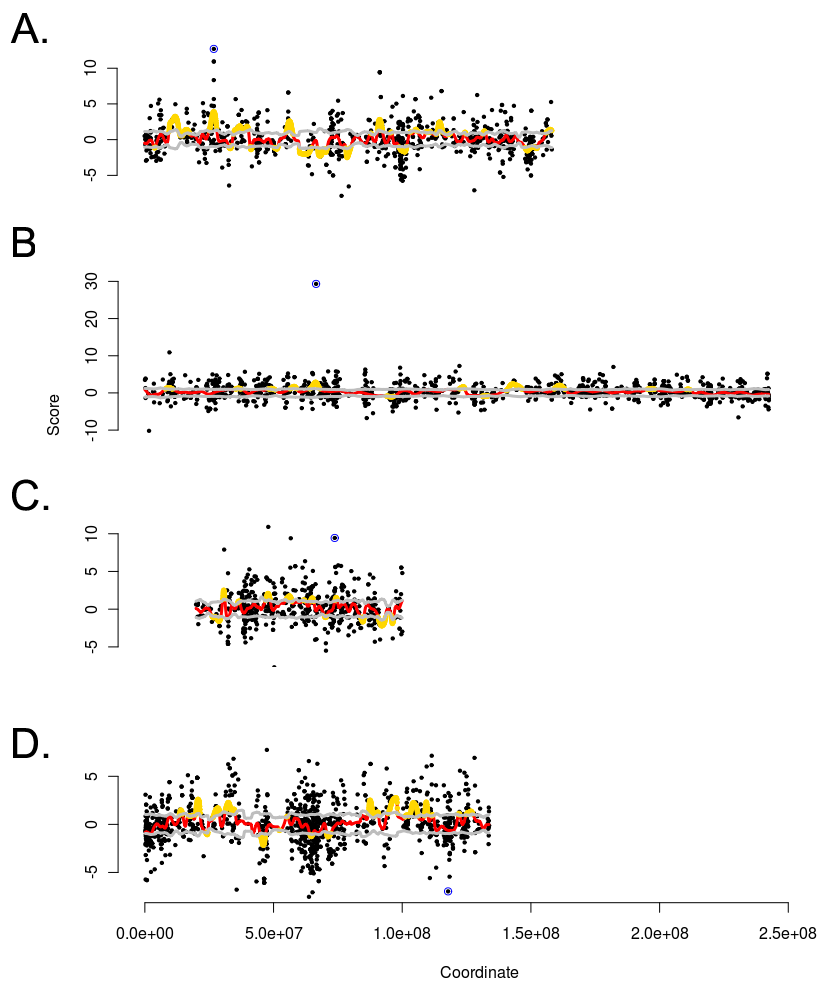


**Figure S6.** Graphical representation of imbalanced regions identified with MACAT in chromosome 7 (A), chromosome 2 (B), chromosome 15 (C) and chromosome 11 (D) using 100 permutation. The red line describes chromosome profile that change color (yellow) in regions statistically imbalanced. Grey lines describe the sliding average of the 0.025 and 0.975 quantiles of the permuted scores. Blue dot are used to indicate HOX cluster on chromosome 7, the region containing Meis homeobox 1 (MEIS1) on chromosome 2, the chondroitin sulfate proteoglycan 4 (CSPG4/NG2) on chromosome 15 and MLL region on chromosome 11. MLL region was not detected. Data refer to leukaemia dataset.


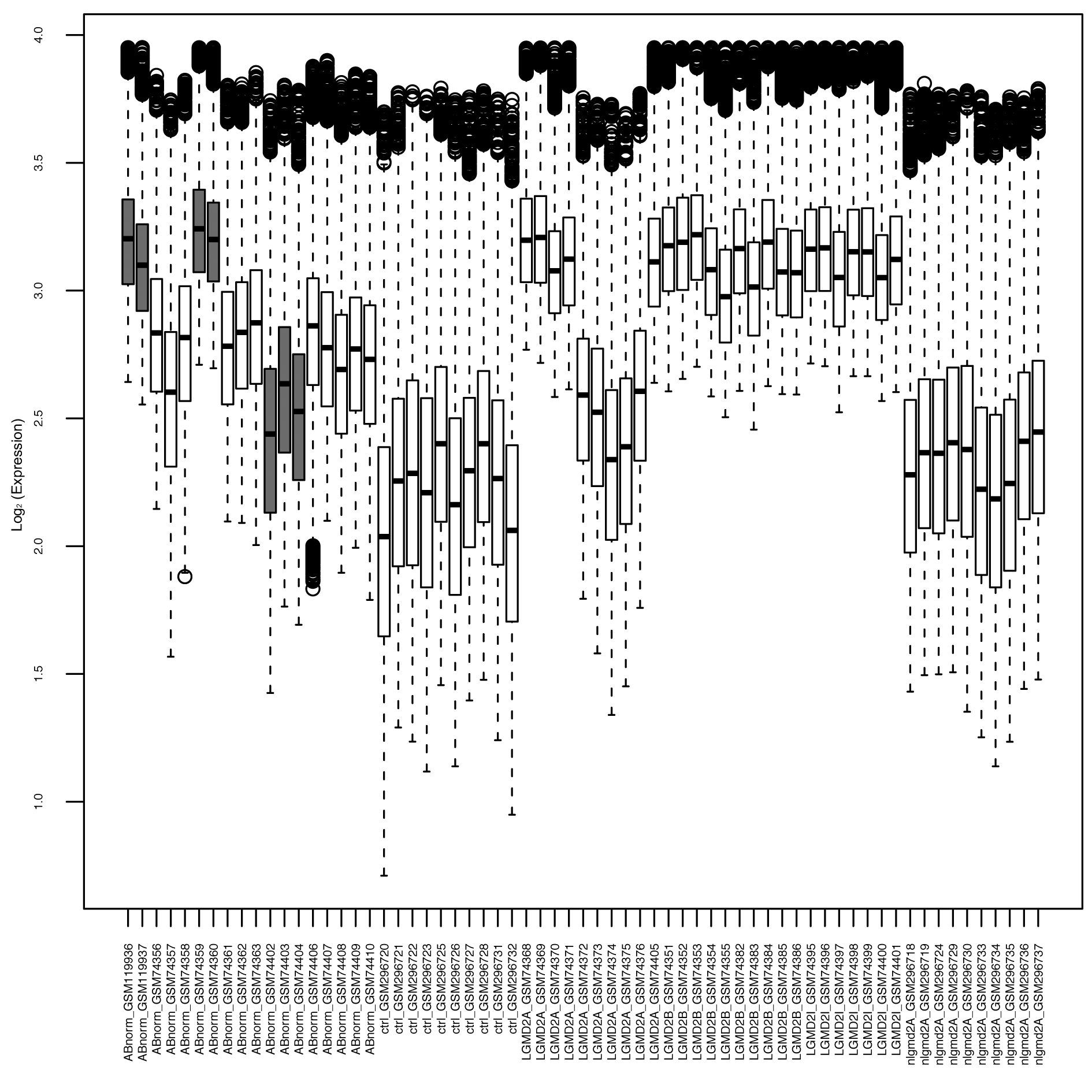


**Figure S7.** Boxplot of raw expression data from LGMDs analysis. Grey boxes indicate controls excluded from analyses.


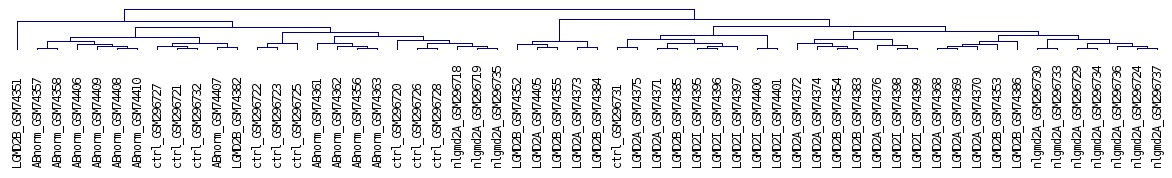


**Figure S8.** Unsupervised cluster analysis on ALL significant (FDR ≤ 0) differentially expressed genes calculated with SAM analysis (LGMD2A vs CTRLs).
